# An Assessment of the Impacts of Feeding Four Fungal Extracts on the Lifespan and Midgut of Newly Emerged Carniolan Honey Bees (*Apis mellifera carnica*)

**DOI:** 10.3390/insects17060594

**Published:** 2026-06-05

**Authors:** Leticia S. Ansaloni, Caio E. C. Domingues, Marija Gregori, Andrej Gregori, Aleš Gregorc

**Affiliations:** 1 Faculty of Agriculture and Life Sciences, University of Maribor, Pivola 10, 2311 Hoče, Slovenia; cecdomingues@gmail.com (C.E.C.D.); ales.gregorc@um.si (A.G.); 2 MycoMedica Ltd., Podkoren 72, 4280 Kranjska Gora, Slovenia; marija.gregori@zanaravo.com (M.G.); andrej.gregori@zanaravo.com (A.G.); 3 Biotechnical Faculty, University of Ljubljana, Jamnikarjeva 101, 1000 Ljubljana, Slovenia

**Keywords:** bee health, complementary feed, fungi, histoarchitecture, midgut morphology

## Abstract

Given the current global agricultural system, honey bees are exposed to a complex network of stressors that can act simultaneously, making it challenging to maintain healthy colonies. Therefore, studies on natural products to improve colony health have increased in recent years. Among them, fungal extracts have been shown to be beneficial to honey bees. However, there remains a knowledge gap regarding lifespan and histomorphological studies in bees fed fungal extracts. Our current study aimed to assess the impacts of extracts from *Ganoderma lucidum* (GL), *Hericium erinaceus* (HE), *Inonotus obliquus* (IO), and *Trametes versicolor* (TV) on the lifespan and midgut of honey bees. Newly emerged Carniolan honey bees (*Apis mellifera carnica*) were fed 4% of each fungal extract until the death of the last individual to assess survival probability. For histomorphological analyses, bees were fed for 7, 14, and 21 days and sampled at these same time points. Then, the midguts were dissected and histologically processed for qualitative and semi-quantitative microscopic analyses. The results showed that the fungal extracts did not significantly affect honey bee survival, and that the histomorphology of the intestinal villi, digestive cells, and regenerative cells in bees treated with fungal extracts did not differ from that of untreated bees throughout the analyzed period. Similarly, no differences were observed in the midgut lesion index between bees treated with fungal extracts and the untreated group. Overall, the absence of harmful effects on lifespan and midgut suggests that feeding fungal extracts may be a potential alternative for supporting bee health.

## Simple Summary

Honey bees are important pollinators for food production and natural ecosystems, but they continue to face health threats from multiple stressors. In order to support bee health and maintain healthy colonies, natural products such as fungal extracts have been investigated as potential supplementary feed. However, there is still a knowledge gap regarding their safety. In this study, honey bees were fed extracts from four fungi (*Ganoderma lucidum*, *Hericium erinaceus*, *Inonotus obliquus*, and *Trametes versicolor*), and survival probability and midgut damage were assessed over the experimental period. Our results showed that the fungal extracts tested did not reduce honey bee survival probability or cause harmful changes in the midgut. These findings, in agreement with the recent literature, suggest that fungal extracts may represent a safe natural alternative for supporting honey bee health.

## 1. Introduction

Honey bees (*Apis mellifera*, Linnaeus 1758) are recognized as important pollinators in agricultural systems and natural habitats [1,2], as well as due to the commercialization of their hive products [3]. However, due to widespread reports of honey bee colony decline across several continents [4,5,6,7,8,9], together with the multiple stressors to which they are exposed [10], maintaining healthy colonies has become more challenging over the years. According to French et al. [11], honey bees can be exposed to multiple stressors simultaneously, which may vary depending on the crop type and geographic region, increasing the complexity of factors affecting colony health.

In this context, natural products used as complementary feed may represent a promising approach to maintaining strong, resilient, and healthy honey bee colonies [12]. Several studies have reported the positive effects of such supplements on honey bees [12,13,14,15,16,17]. Yeast-supplemented diets can maintain survival and fat body accumulation in honey bees, in addition to keeping immune-related genes within an optimal range of expression [18]. Cannabidiol oil derived from hemp extracts has been shown to stimulate the antioxidant system in honey bee workers [16]. Piperine-treated honey bee workers showed increased lifespan, protein concentration, and antioxidant enzyme activities compared to the control group [13]. Leaf ethanolic extracts of bay laurel (*Laurus nobilis*) reduced the viral load and replication of black queen cell virus (BQCV) in honey bee foragers [19], showing the promising potential of natural products.

Among the high diversity of natural products, several fungi have attracted attention due to their bioactive compounds and potential health-related applications. *Ganoderma lucidum* is a well-known mushroom that is commercially exploited in different forms (e.g., beverages, cosmetics, medicinal products, dietary supplements, and food), and whose medicinal properties, linked to its bioactive compounds, have been extensively studied [20,21,22,23]. The principal bioactive compounds include polysaccharides, triterpenoids, proteins and peptides, and sterols, as well as other constituents such as vitamins and mineral elements [24]. In addition to the potentially beneficial effects on human health reported in the literature [25], studies have also suggested that *G. lucidum* may promote animal health [26,27,28].

Similarly, *Hericium erinaceus* is an edible mushroom with numerous bioactive compounds, such as polysaccharides, proteins, lectins, phenols, and terpenoids, which has attracted attention due to its potential applications in commercial products (e.g., alternative medicines, dietary supplements, and food products) [29,30,31]. A toxicological assessment showed no adverse effects on hematological, biochemical, or histopathological (liver, kidney, brain, heart, lung, and spleen) parameters in Sprague-Dawley rats fed *H. erinaceus* at doses of 250, 500, and 1000 mg/kg body weight for 90 days [32], indicating normal physiological function and the maintenance of animal health throughout the study period and suggesting a favorable safety profile for consumption.

*Inonotus obliquus* is also known as a medicinal fungus because it contains bioactive compounds such as lanostane-type triterpenoids, melanin, lignin-derived compounds, and polysaccharides, which have been associated with potential anticancer, anti-inflammatory, antiviral, antioxidant, hypoglycemic, and other biological activities [33,34,35,36,37]. According to Diao et al. [38], *I. obliquus* showed potential benefits against diabetes in male adult Wistar rats. However, although the pharmacological potential of *I. obliquus* has been extensively studied and the results are promising, further scientific rigor and standardization are still required [39].

In the same context, *Trametes versicolor* is an edible mushroom with physiologically bioactive compounds such as β-glucan polysaccharides, triterpenoids, sterols, proteins, peptides, and bioactive sphingolipids, with medicinal applications [40,41,42]. β-Glucans from *T. versicolor* were reported to enhance survival rates and reduce lung viral titers in chickens infected with the H9N2 avian influenza virus [43]. Furthermore, the industrial applications of *T. versicolor*, including biofuels, biofertilizers, feeds, biotransformation processes, and wastewater treatment have been considered, although their potential has not yet been fully explored [44].

The biological activities of fungal compounds have also attracted attention in apicultural research. Fungal extracts have shown potential against the viruses deformed wing virus (DWV) and Lake Sinai virus (LSV), reducing their levels in honey bees in a dose-dependent manner [45]. Extracts of *Agaricus bisporus* improved the survival and immunity of honey bees infected with *Nosema ceranae* [46]. According to Parish et al. [47], pollen supplemented with fungal spores can increase the longevity of honey bee workers, which may indicate the nutritional value of these spores. Extracts from *G. lucidum*, *H. erinaceus*, *I. obliquus*, and *T. versicolor* increased immune responses in Carniolan honey bees fed for 7, 14, and 21 days [48].

However, although the use of fungal extracts has shown promising results on honey bees health, there are a lack of studies on their effects on honey bee lifespan and organs, particularly with respect to gut morphology and integrity. The honey bee digestive system is divided into the foregut, midgut, and hindgut [49,50]. The foregut is responsible for the ingestion, transportation, and storage of food, the midgut for the absorption and digestion of nutrients, and the hindgut for reabsorption and excretion [50,51]. Due to its function, the midgut represents a valuable and well-established tool to analyze morphological changes according to severity and reversibility [52]. Several studies have used the midgut as a morphological marker to assess structural changes [53,54,55,56,57,58,59,60]. Thus, the midgut can be a sensitive indicator of dietary effects on honey bee health.

Therefore, given the potential importance of fungal extracts for honey bee health and the current lack of studies addressing their effects on the midgut, the present study aimed to evaluate the effects of supplementary feeding with extracts of *G. lucidum*, *H. erinaceus*, *I. obliquus*, and *T. versicolor* on lifespan and midgut histological lesions in *A. m. carnica* (Pollmann, 1879).

## 2. Materials and Methods

### 2.1. Fungal Extracts

In this study, four fungal extracts (*G. lucidum*, *H. erinaceus*, *I. obliquus*, and *T. versicolor*) were produced by MYCOMEDICA (MycoMedica Ltd., Podkoren, Slovenia), where the cultivation of axenic cultures, the preparation of substrate media, and the production of fungal extracts were carried out according to the methodology described by Ansaloni et al. [48]. The extracts used in this study were ethanolic (50%), and no additional solvents or emulsifiers were employed during the preparation of the food used in the experiments.

### 2.2. Honey Bee Sampling

An apiary with ten colonies of Carniolan honey bees (*A. m. carnica*) located in the Botanical Garden of the University of Maribor (46°30′16.5″ N 15°38′01.2″ E), in the municipality of Hoče (Slovenia), was used in this study. Only colonies of similar strength which were visually asymptomatic and free of parasites were used to perform the experiments. In order to obtain newly emerged workers, one capped brood frame was taken from each of the three different colonies and transported in a wooden nucleus hive (43 × 23 cm; height 29.5 cm) to the laboratory at the Faculty of Agriculture and Life Sciences. The capped brood frames were placed inside an incubator at 35 °C (±1 °C) and 70% (±5%) relative humidity, in darkness, for the emergence of the workers.

### 2.3. Experimental Assay

As soon as the workers emerged, honey bees up to 24 h old from three colonies were placed into bee cages (plastic cups, 7.5 cm in diameter × 12.5 cm in height) containing 3 mm diameter aeration holes across the entire surface and a feeder (1.5 mL plastic microtube) with syrup solution (50% water, 50% sucrose) provided ad libitum. The bees were kept for an acclimation period of 24 h inside an incubator at 31 °C (±1 °C) and 70% (±5%) relative humidity, in darkness. The bee cages were covered with a plastic Petri dish (85 mm in diameter) containing filter disk paper. After the acclimation period of 24 h, the feeders containing syrup solution were removed six hours prior to the start of the experiment, and the newly emerged bees were randomly assigned to the experimental groups and maintained under the same conditions as during acclimation (31 °C ± 1 °C, 70% ± 5% relative humidity, and darkness) as follows: *G. lucidum* (GL), *H. erinaceus* (HE), *I. obliquus* (IO), and *T. versicolor* (TV), each at a concentration of 4% (*v*/*v*) in syrup, along with an untreated control group (CTL), which received only syrup (50% water, 50% sucrose). For each experimental group, five bee cages containing 20 bees each were used, totaling 100 bees per treatment, and two experiments were performed in this study. The first experiment was conducted to determine survival probabilities. Bees were fed ad libitum, and mortality was recorded daily throughout the experimental period. In the second experiment, bees were fed ad libitum for up to 21 days, and individuals were sampled after 7, 14, and 21 days for subsequent histomorphological analyses. In both experiments, feeders were refilled daily with fresh solution.

The concentration of the fungal extracts were defined based on previous experiments conducted by our research group and Ansaloni et al. [48]. The methodology followed the guidelines outlined in the Organization for Economic Co-operation and Development Test No. 245 [61], with adaptations to the number of bees, temperature, starvation period, and exposure period.

### 2.4. Histopathological Processing

Ten bees from each experimental group were sampled after 7, 14, and 21 days, and their intestines were dissected at room temperature under a stereomicroscope (Leica EZ4 W, Leica Microsystems (Schweiz) AG, Heerbrugg, Switzerland). After dissection, the midguts were individually placed in plastic microtubes (1.5 mL capacity) containing a fixative solution (4% paraformaldehyde in 0.1 mol L^−1^ phosphate buffered saline: PBS, pH 7.4) at a volume ten times greater than that of the organs, for up to 24 h. The organs were then washed twice with PBS for 30 min and dehydrated with ethanol solutions (30%, 50%, 60%, 70%, 85%, 90%, 95%, and 100%) at 4 °C for 1 h in each solution [62,63]. After the dehydration step, midguts were individually immersed in the infiltration solution (50 mL of basic resin liquid + 1 packet of 0.5g activator) to ensure complete penetration and then embedded (infiltration solution + hardener) following all the manufacturer’s instructions of the HISTORESIN Embedding Kit (Leica Biosystems Nussloch GmbH, Nussloch, Germany). After complete polymerization, the resin blocks containing the midguts in HistoMold (6 × 8 mm, Leica) were glued onto wooden blocks (5 mm in height, 28 mm in width, and 40 mm in length), and histological sections of 6 µm were obtained using an automated rotary microtome, the HistoCore AUTOCUT R (Leica Biosystems). The histological sections were placed in a floating bath (Bio Optica Milano Spa, Milano, Italy) at 40 °C and subsequently mounted on slides. They were stained with hematoxylin and eosin and coverslipped using DPX Mountant (Sigma-Aldrich, Saint Louis, MO, USA) for histological study by light microscopy using an Olympus BX51TF (Olympus Corporation, Tokyo, Japan).

### 2.5. Qualitative Characterization of Midguts

A qualitative analysis of the midguts of all experimental groups was performed as described by Domingues et al. [57]. Five individuals per experimental group at each time point (7, 14, and 21 days) were used in the analyses (*n* = three slides per individual, with 12 histological sections per slide). A total of 180 non-sequential histological sections per experimental group were analyzed at each time point by double-blind method, and 90 images of the midgut were acquired to study midgut morphology and cellular changes per experimental group at each time point using OLYMPUS cellSens software (Entry 1.16, build 15404).

### 2.6. Semi-Quantitative Characterization of Midguts

Semi-quantitative analyses were performed using images obtained during the qualitative midgut analyses described above. To determine the midgut lesion index, these analyses were conducted following protocols adapted for bees that consider both the severity (pathological importance) and reversibility (level of intensity) of morphological changes [52,57,64]. Lesions were classified according to their pathological importance factor (PIF): level 1—reversible (eliminated cell, apocrine secretion, and spherocrystals); level 2—moderately reversible (atypical nuclei, and cell vacuolization); level 3—irreversible (cell pyknosis, and the alteration of nests of regenerative cells). Lesions were also graded based on their extent of occurrence as follows: 0—no occurrence; 1—minor occurrence; 2—moderate occurrence; and 3—severe occurrence. A lesion index was calculated for each parameter, and the total organ lesion index was obtained through the summing of recoded lesion indexes [52,57,64,65].

### 2.7. Statistical Analysis

Survival probability data were analyzed using the Log-rank (Kaplan–Meier) test, followed by multiple comparisons among all experimental groups using the Holm–Sidak method. The data obtained from the semi-quantitative analysis of the midguts were first evaluated using the Kolmogorov–Smirnov and Shapiro–Wilk normality tests, followed by the Kruskal–Wallis non-parametric test with Dunn’s post hoc test for multiple comparisons. Statistical analyses were performed using GraphPad Prism 11.0.1 (90) (GraphPad Software, LLC, San Diego, CA, USA), adopting a significance level of *p* < 0.05. The graphs are presented as mean ± standard error.

## 3. Results

**Figure 1:**
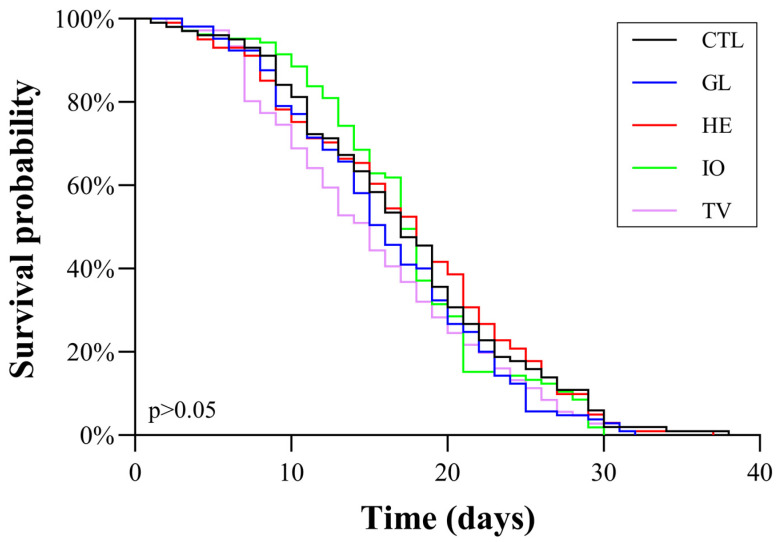
The survival probability of newly emerged Carniolan honey bee workers (*A. m. carnica*) fed fungal extracts. Untreated control group (CTL), *G. lucidum* (GL), *H. erinaceus* (HE), *I. obliquus* (IO), and *T. versicolor* (TV). *n* = 100 honey bee workers per experimental group. Survival analysis (Kaplan–Meier) showed no significant differences among groups (log-rank test, chi square = 6.611, df = 4, *p* = 0.1579), followed by multiple comparisons using the Holm–Sidak method.

The survival probability experiment, in which honey bees were fed four fungal extracts ad libitum, lasted 38 days and ended when the last individual died. The results showed that fungal feeding did not significantly affect honey bee survival (*p* > 0.05), and the mean survival times were similar across all experimental groups (Figure 1). Bees from the CTL group had a mean survival of 17 days (95% confidence interval: 15–19 days), while those from the GL group showed a mean survival of 16 days (95% confidence interval: 14–18 days). Bees from the HE group presented a mean survival of 18 days (95% confidence interval: 16–20 days), whereas bees from the IO group exhibited a mean survival of 17 days (95% confidence interval: 17–18 days), and bees from the TV group had a mean survival of 15 days (95% confidence interval: 12–16 days).

**Figure 2:**
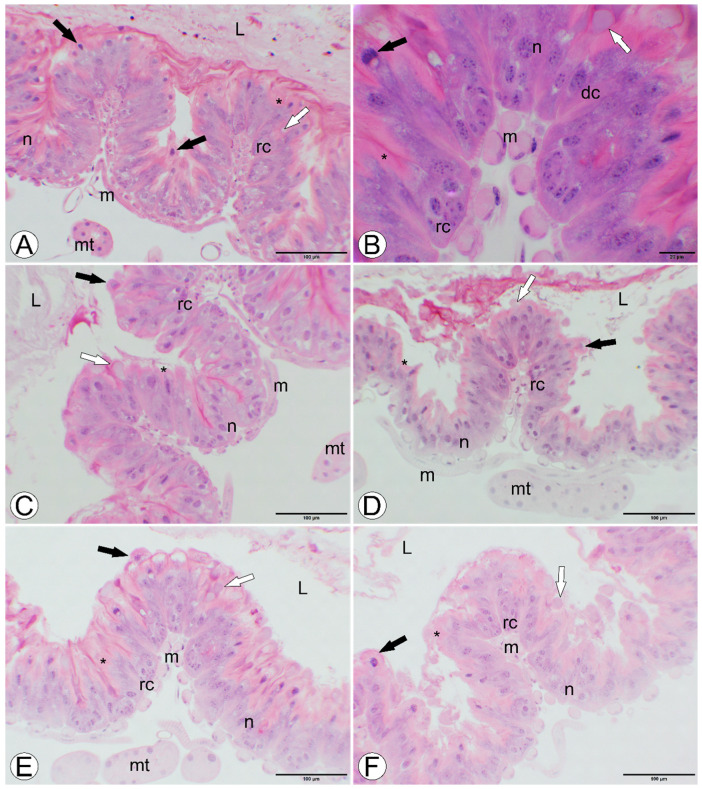
Midgut of 7-day-old Carniolan honey bees (*A. m. carnica*) following a seven-day feeding period with fungal extracts. (**A**,**B**) Untreated control group (CTL), (**C**) *G. lucidum* (GL), (**D**) *H. erinaceus* (HE), (**E**) *I. obliquus* (IO), and (**F**) *T. versicolor* (TV). Asterisk = brush border, black arrow = cell being eliminated, dc = digestive cell, L = lumen, m = muscle, mt = Malpighian tubule, n = nuclei, rc = nest of regenerative cells, and white arrow = apocrine secretion. Histological sections were stained with hematoxylin-eosin. *n* = five individuals per experimental group. (**A**,**C**–**F**) Bars = 100 µm. (**B**) Bar = 20 µm.

The histological structure of the midgut of *A. m. carnica* worker bees, after 7 days of feeding, showed intact intestinal villi, digestive cells (enterocytes), a visible brush border along the apical surface of the cells, and visceral muscle fibers across all fungal extract treatments (GL, HE, IO, and TV), with morphology similar to that of the CTL group (Figure 2). A similarly well-organized morphology, in the form of clusters, was observed in the nests of regenerative cells at the base of the epithelium in all experimental groups, as shown in Figure 2B, representing the typical morphology observed in these groups. Cells being eliminated and apocrine secretion released into the lumen were also observed in all experimental groups (Figure 2A,C–F). Other parameters in the midgut, such as spherocrystals, atypical nuclei, cytoplasmic vacuolization, and pyknosis, were detected at very low frequencies and did not represent the typical pattern of the organ in the experimental groups.

**Figure 3:**
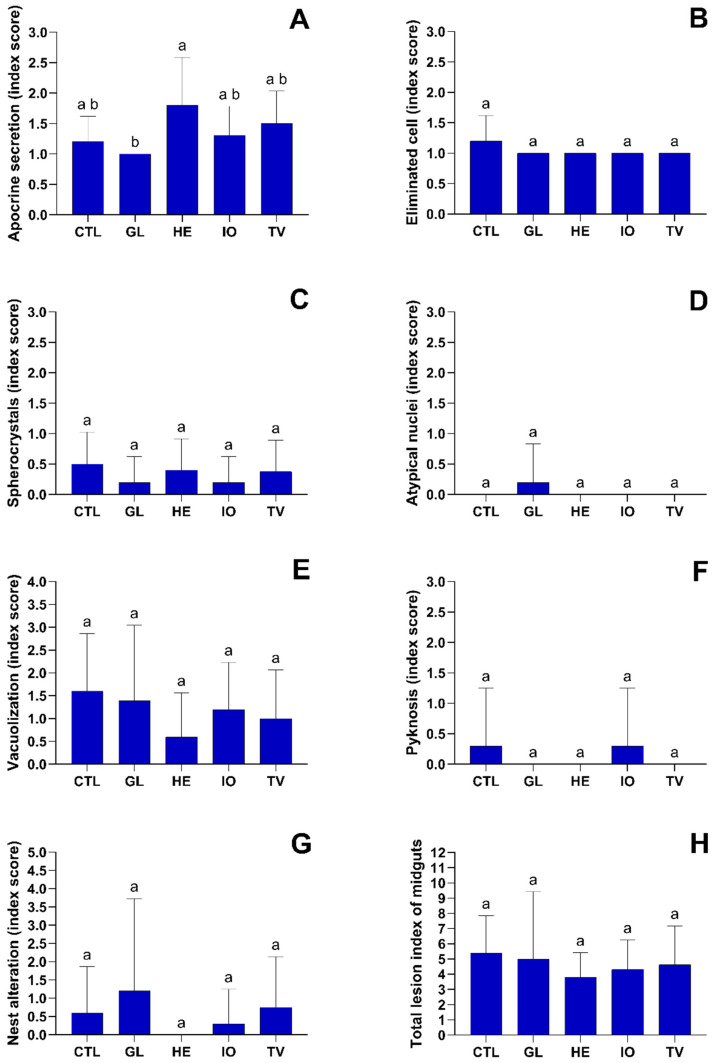
Midgut lesion indices and the total midgut lesion index of 7-day-old Carniolan honey bees (*A. m. carnica*) following a seven-day feeding period with fungal extracts. Untreated control group (CTL), and fungal extract treatments: *G. lucidum* (GL), *H. erinaceus* (HE), *I. obliquus* (IO), and *T. versicolor* (TV). (**A**) Apocrine secretion, (**B**) eliminated cell, (**C**) spherocrystals, (**D**) atypical nuclei, (**E**) vacuolization, (**F**) pyknosis, (**G**) nest alteration, and (**H**) total lesion index of midguts. Different lower-case letters denote significant statistical differences determined by the Kruskal–Wallis test followed by Dunn’s post hoc test (*p* < 0.05). *n* = five individuals per experimental group.

Regarding the semi-quantitative characterization of the midguts of *A. m. carnica* worker bees after 7 days of feeding, no significant changes in apocrine secretion (PIF level 1) were observed in any experimental group compared to the CTL group (*p* > 0.05) (Figure 3A). However, a statistically significant difference (*p* < 0.05) was found between the HE group, which exhibited the highest mean index score (1.8), and the GL group, which exhibited the lowest mean index score (1.0) (Figure 3A). No significant changes were noted among experimental groups (*p* > 0.05) for eliminated cells (PIF level 1, Figure 3B), spherocrystals (PIF level 1, Figure 3C), atypical nuclei (PIF level 2, Figure 3D), vacuolization (PIF level 2, Figure 3E), pyknosis (PIF level 3, Figure 3F), or nest alterations (PIF level 3, Figure 3G). The total lesion index of midguts (Figure 3H), calculated as the sum of all lesions classified according to the PIF, had mean values of 5.4 for the CTL group, 5.0 for GL, 3.8 for HE, 4.3 for IO, and 4.6 for TV, with no statistically significant differences among the experimental groups (*p* > 0.05).

**Figure 4:**
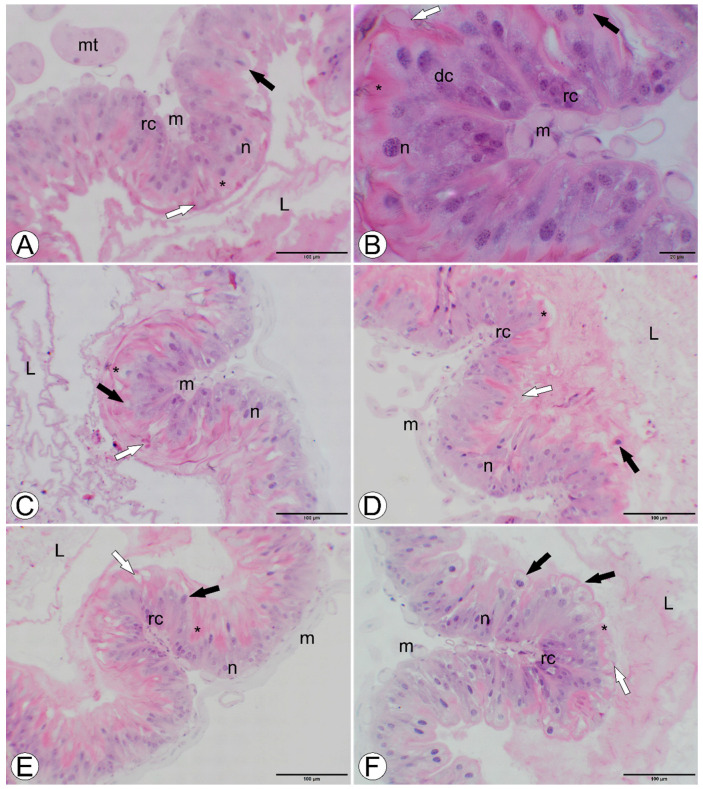
Midgut of 14-day-old Carniolan honey bees (*A. m. carnica*) following a fourteen-day feeding period with fungal extracts. (**A**,**B**) Untreated control group (CTL), (**C**) *G. lucidum* (GL), (**D**) *H. erinaceus* (HE), (**E**) *I. obliquus* (IO), and (**F**) *T. versicolor* (TV). Asterisk = brush border, black arrow = cell being eliminated, dc = digestive cell, L = lumen, m = muscle, mt = Malpighian tubule, n = nuclei, rc = nest of regenerative cells, and white arrow = apocrine secretion. Histological sections were stained with hematoxylin-eosin. *n* = five individuals per experimental group. (**A**,**C**–**F**) Bars = 100 µm. (**B**) Bar = 20 µm.

Following 14 days of feeding, similar to the results observed after 7 days (Figure 2), no changes were detected in the midgut histological structure of *A. m. carnica* worker bees across all experimental groups (Figure 4). The morphological pattern of the intestinal villi in the fungal extract treatments (GL, HE, IO, and TV) was similar among treatments and to the CTL group, including the presence of a visible brush border at the apical surface of the cells, visceral muscle fibers, as well as cells elimination and apocrine secretion into the lumen (Figure 4A,C–F). The nests of regenerative cells exhibited similar morphological patterns across all experimental groups and were clearly visible in clusters at the base of the epithelium, surrounded by digestive cells (enterocytes) (Figure 4B). The similar parameters, observed at a low frequency after seven days of feeding with fungal extracts (spherocrystals, atypical nuclei, and cytoplasmic vacuolization), were also detected after 14 days, with only a minor occurrence in the experimental groups. No pyknosis was observed in the cells.

**Figure 5:**
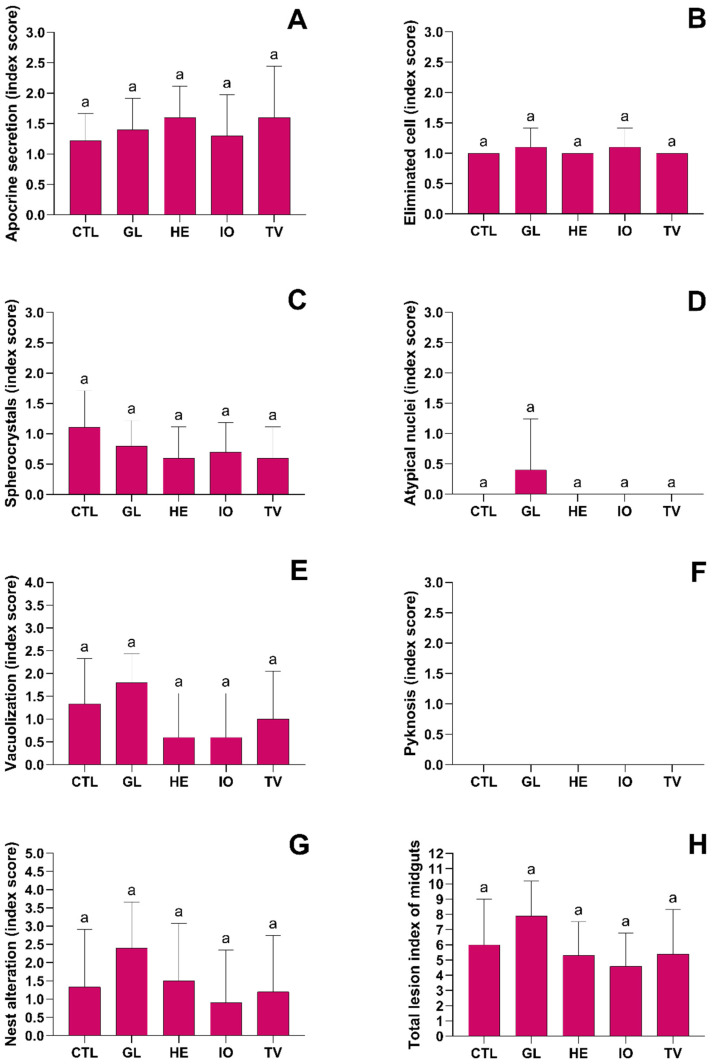
Midgut lesion indices and total midgut lesion index of 14-day-old Carniolan honey bees (*A. m. carnica*) following fourteen-day feeding period with fungal extracts. Untreated control group (CTL), and fungal extract treatments: *G. lucidum* (GL), *H. erinaceus* (HE), *I. obliquus* (IO), and *T. versicolor* (TV). (**A**) Apocrine secretion, (**B**) eliminated cell, (**C**) spherocrystals, (**D**) atypical nuclei, (**E**) vacuolization, (**F**) pyknosis, (**G**) nest alteration, and (**H**) total lesion index of midguts lesion index. Different lower-case letters denote significant statistical differences determined by Kruskal–Wallis test followed by Dunn’s post hoc test (*p* < 0.05). *n* = five individuals per experimental group.

The semi-quantitative characterization of the midguts of *A. m. carnica* worker after 14 days of feeding is shown in Figure 5. No significant changes were observed in any of the analyzed parameters, including apocrine secretion (PIF level 1, Figure 5A), eliminated cells (PIF level 1, Figure 5B), spherocrystals (PIF level 1, Figure 5C), atypical nuclei (PIF level 2, Figure 5D), vacuolization (PIF level 2, Figure 5E), pyknosis (PIF level 3, Figure 5F), and nest alteration (PIF level 3, Figure 5G) when compared to bees from the CTL group (*p* > 0.05). Pyknosis was not detected in any of the experimental groups (PIF level 3, Figure 5F). The total lesion index of midguts (Figure 5H) had mean values of 6.0 for the CTL group, 7.9 for GL, 5.3 for HE, 4.6 for IO, and 5.4 for TV, with no statistically significant differences among the experimental groups (*p* > 0.05).

**Figure 6:**
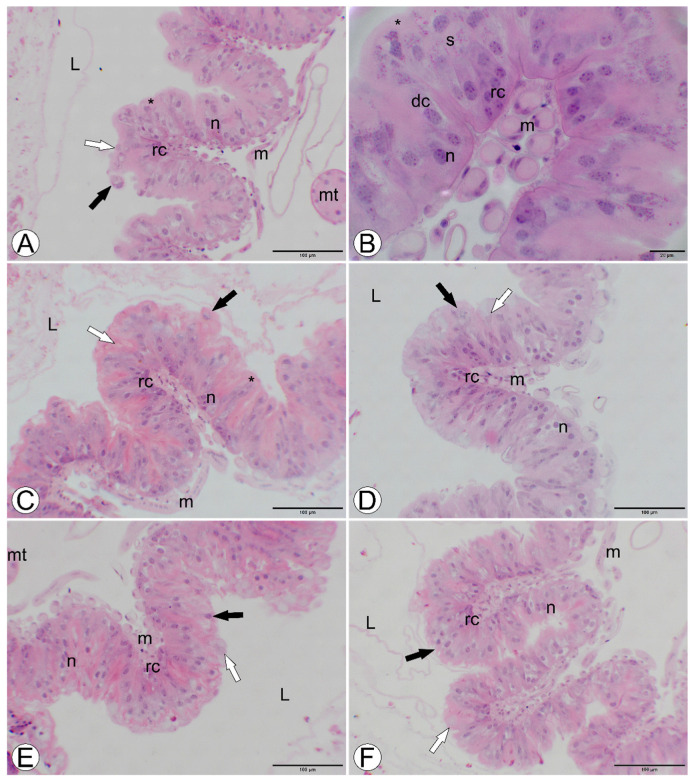
Midgut of 21-day-old Carniolan honey bees (*A. m. carnica*) following twenty-one-day feeding period with fungal extracts. (**A**,**B**) Untreated control group (CTL), (**C**) *G. lucidum* (GL), (**D**) *H. erinaceus* (HE), (**E**) *I. obliquus* (IO), and (**F**) *T. versicolor* (TV). Asterisk = brush border, black arrow = cell being eliminated, dc = digestive cell, L = lumen, m = muscle, mt = Malpighian tubule, n = nuclei, rc = nest of regenerative cells, s = spherocrystals, and white arrow = apocrine secretion. Histological sections were stained with hematoxylin-eosin. *n* = five individuals per experimental group. (**A**,**C**–**F**) Bars = 100 µm. (**B**) Bar = 20 µm.

At the end of the 21-day feeding period with fungal extracts, the histological structure of the midgut was still preserved in *A. m. carnica* worker bees across all experimental groups (Figure 6). The pattern of intestinal villi, including visceral muscle fibers, in the fungal extract treatments (GL, HE, IO, and TV) was similar to that of the CTL group (Figure 6A,C–F). The presence of a cluster of regenerative cells at the basal membrane of the epithelium was evident and similar in all the experimental groups (Figure 6B), but slightly reduced compared to the other analyzed periods. In addition, spherocrystals were more apparent after 21 days, especially in bees from the CTL group (Figure 6A,B). As in the other analyzed periods, a brush border was visible at the apical region of the cells in the fungal extract treatments (GL, HE, IO, and TV), similar to the CTL group (Figure 6). All the experimental groups showed cell elimination and apocrine secretion into the lumen (Figure 6A,C–F), with apocrine secretion being slightly more pronounced in the IO group (Figure 6E), based on the qualitative characterization. Atypical nuclei and cytoplasmic vacuolization were observed at very low frequency, and pyknosis was not detected in any of the experimental groups.

**Figure 7:**
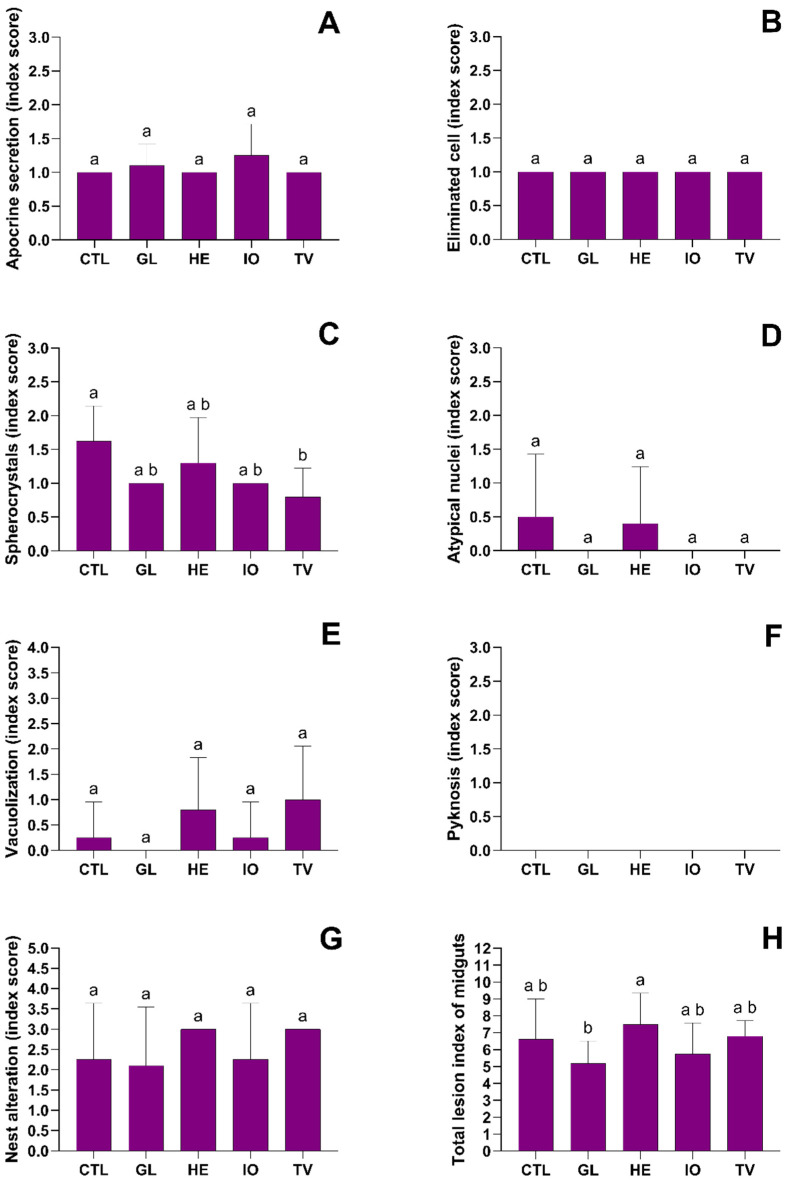
Midgut lesion indices and total midgut lesion index of 21-day-old Carniolan honey bees (*A. m. carnica*) following twenty-one-day feeding period with fungal extracts. Untreated control group (CTL), and fungal extract treatments: *G. lucidum* (GL), *H. erinaceus* (HE), *I. obliquus* (IO), and *T. versicolor* (TV). (**A**) Apocrine secretion, (**B**) eliminated cell, (**C**) spherocrystals, (**D**) atypical nuclei, (**E**) vacuolization, (**F**) pyknosis, (**G**) nest alteration, and (**H**) total lesion index of midguts lesion index. Different lower-case letters denote significant statistical differences determined by Kruskal–Wallis test followed by Dunn’s post hoc test (*p* < 0.05). *n* = five individuals per experimental group.

As shown in Figure 7, the semi-quantitative characterization of the midguts of *A. m. carnica* worker bees at the final experimental period (21 days) following feeding with fungal extracts is presented. No significant changes were observed in apocrine secretion (PIF level 1, Figure 7A), eliminated cells (PIF level 1, Figure 7B), atypical nuclei (PIF level 2, Figure 7D), vacuolization (PIF level 2, Figure 7E), pyknosis (PIF level 3, Figure 7F), which was absent in all treatments, or nest alteration (PIF level 3, Figure 7G) compared to bees from the CTL group (*p* > 0.05). However, a statistically significant difference (*p* < 0.05) in spherocrystals (PIF level 1) was detected between the CTL group (mean index score: 1.6) and the TV group (mean index score: 0.8), whereas the other experimental groups did not differ from the CTL group (*p* > 0.05), as highlighted in Figure 7C. Regarding the total lesion index of midguts (Figure 7H), a statistically significant difference (*p* < 0.05) was observed between the GL group, which exhibited the lowest lesion index score (5.2), and the HE group, which exhibited the highest lesion index score (7.5). The mean values for the other groups were 6.6 for CTL, 5.8 for IO, and 6.8 for TV, and no statistically significant differences were detected among these fungal extract treatments compared to the CTL group (*p* > 0.05).

## 4. Discussion

According to the present results, feeding newly emerged Carniolan honey bees (*A. m. carnica*) with complementary feed containing 4% of each fungal extract (*G. lucidum*, *H. erinaceus*, *I. obliquus*, and *T. versicolor*) did not reduce lifespan or induce alterations in the midgut histoarchitecture or increase midgut lesion indices after 7, 14, and 21 days. While studies addressing the effects of fungal extracts on honey bee organs remain scarce, previous research has reported promising effects on lifespan and immune responses [48], as well as efficacy against microsporidia [46] and viruses [45]. Furthermore, exposure to spores of *Botrytis cinerea*, *Cladosporium* sp., and *Colletotrichum acutatum* has also been associated with nutritional benefits (increased longevity) in honey bee workers, whereas no alterations (normal morphology) were observed in the hypopharyngeal glands and ovaries [47].

A study by Ansaloni et al. [48] reported a novel effect of extracts of *G. lucidum*, *H. erinaceus*, *I. obliquus*, and *T. versicolor*, each at a 4% concentration, which increased antioxidant activity, total antioxidant capacity, and metabolic marker levels in Carniolan honey bee (*A. m. carnica*) foragers after 14 and 21 days of feeding, with *I. obliquus* producing the highest levels among the markers studied. Additionally, *G. lucidum* extract increased honey bee longevity by 16.4% compared to the control group. Similarly, the present results indicate that feeding newly emerged honey bees with the same fungal extracts at the same concentration did not induce harmful effects in the midgut throughout the experimental period, thereby supporting the safety profile of these fungal extracts in honey bees. However, in contrast to the findings reported by Ansaloni et al. [48], no increase in longevity (*p* > 0.05) was observed in newly emerged bees in the present study (Figure 1). This finding may be associated with physiological and metabolic differences between newly emerged workers and forager bees. According to Bonilla-Rosso and Engel [66], gut microbiota also impacts bee health and physiology, which may influence nutrient absorption, immune responses, and fitness factors, as the composition of bee gut microbiota can vary depending on several factors (e.g., season, diet, host age, among others).

The absence of alterations in the midgut histoarchitecture of Carniolan honey bee (*A. m. carnica*) workers after 7, 14, and 21 days of feeding (Figure 2, Figure 4 and Figure 6) may be related to the bioactive compounds (e.g., polysaccharides, terpenoid/triterpenoid compounds, proteins, and phenolic constituents) present in the fungal extracts evaluated in this study, which have been associated with various biological activities [20,24,28,29,31,34,36,42,43]. Additionally, a similar absence of effects has also been reported in the liver and kidney tissues of Wistar rats fed *G. lucidum* [67], as well as in the liver, kidney, brain, heart, lung, and spleen of Sprague-Dawley rats fed *H. erinaceus* [32]. A study by Ishfaq et al. [68] showed that an aqueous extract of *I. obliquus* prevented the microcystin-induced histological alterations associated with hepatotoxicity in mice, suggesting its potential use as a supplement in the prevention of liver toxicity and inflammation. In the same way, a polysaccharide extract from *T. versicolor* showed wound healing and tissue repair effects in mice with excision wounds [69].

In this context, based on the recent literature, our findings suggest that the tested fungal extracts may be non-toxic to honey bees under the conditions evaluated and could be beneficial, as highlighted by Ansaloni et al. [48]. Nevertheless, studies using natural products and applying lesion index approaches in honey bees are still limited, since this methodology has been mainly applied to pesticide exposure [52,57,64]. Given that the midgut is an important target for histopathological assessment, lesion index scoring provides a quantitative measure of structural alterations and tissue integrity under different experimental conditions [52].

The midgut plays a key role in the enzymatic digestion of food and nutrient absorption in the honey bee digestive system [51]. The midgut epithelium of adult honey bees is composed of three cellular types, digestive, regenerative, and endocrine cells, with distinct functions [50,51]. Digestive cells constitute the majority of the cells in the midgut epithelium and are responsible for synthesizing digestive enzymes, producing components of the peritrophic matrix, which acts as a physical and biochemical barrier against pathogens, toxins, and mechanical damage in the intestine, and absorbing nutrients [50,70]. Endocrine cells are involved in hormone production, while regenerative cells are undifferentiated and replace the other two cell types, maintaining epithelial renewal and integrity [50,70]. Due to their physiological importance, alterations in these midgut cells may represent a significant risk to the health of individual bees and consequently compromise colony performance in the long term.

Numerous studies have demonstrated that insecticides [54,56,71,72,73], fungicides [55,57,59,74,75], herbicides [76], and combined stressors [60,77,78] can impair the structural and functional integrity of this tissue in honey bees. These effects are often associated with histopathological changes in the midgut epithelium, indicative of stress in honey bees [53].

Acute oral exposure of newly emerged honey bees (*A. mellifera*) to the insecticide imidacloprid at the LC_50_ (1.44 mg L^−1^) led to midgut damage, characterized by cytoplasmic vacuolization in the epithelium, enlarged intercellular spaces, the disorganization of the striated border, nuclear pyknosis, and also the presence of apical protrusions in digestive cells, with an organ injury index that increased over time (0.5, 1, 3, 6, 12, and 24 h) [72]. A five-day period of oral exposure to the fungicide pyraclostrobin (0.125 ng a.i./mL and 0.025 ng a.i./mL) increased cytoplasmic vacuolization in the digestive cells, as well as in the nest of the regenerative cells at the base of the epithelium of foragers of honey bees (*A. mellifera*), with an organ injury index higher than in control bees [57]. Tiritelli et al. [78] demonstrated that the combination of *N. ceranae* infection, and microplastic and flupyradifurone consumption reduced the number of regenerative cell nests, increased the frequency of nuclei with compacted chromatin, and increased the frequency of cytoplasm vesiculation in the midgut digestive cells of newly emerged honey bees (*A. m. ligustica*). In contrast, our findings showed that oral exposure to fungal extracts (*G. lucidum*, *H. erinaceus*, *I. obliquus*, and *T. versicolor*) did not induce any injury nor affect the total lesion index in the midgut over time (7, 14, and 21 days).

Currently, morphological studies on bees have become more frequently associated with the lesion index through semi-quantitative analysis [52]. The selected indices (PIF level 1: apocrine secretion, eliminated cells, and spherocrystals; PIF level 2: atypical nuclei and vacuolization; PIF level 3: pyknosis and the alteration of nests of regenerative cells) were based on the literature that had previously been adapted for use in studies on bees [52,57,64,79]. However, the present study represents a novel application of this approach to assess the effects of fungal extracts in the midgut of honey bees. The results showed that the parameters studied were not affected by feeding honey bees with fungal extracts and were similar to those observed in the control groups of newly emerged and foragers Africanized honey bees exposed to three concentrations of fungicide pyraclostrobin, as reported by Domingues et al. [57].

Given the absence of harmful effects on the midgut histoarchitecture in this study, together with the previous reports of the beneficial effects of fungal extracts on honey bees [45,46,48] and the bioactive compounds present in the extracts used here, these findings suggest that such extracts could potentially be used as a dietary supplement in beekeeping practices, highlighting their promising role in supporting and enhancing honey bee health. However, it is important to emphasize that further laboratory experiments assessing other organs, as well as colony-level studies, are still required to validate both the safety and efficacy of *G. lucidum*, *H. erinaceus*, *I. obliquus*, and *T. versicolor* for honey bees.

## 5. Conclusions

In summary, the results of this study underscored that feeding newly emerged honey bees with fungal extracts did not affect lifespan, nor affect the histological structure of the midgut or increase the lesion index after seven, fourteen, and twenty-one days. Considering the importance of bees as pollinators and the multiple stressors they are exposed to, further studies of this topic are necessary to address the current knowledge gap concerning the effects of fungal extracts on bee organs and explore their potential as a complementary feed to support bee health and individual bee and honey bee colony development.

## Data Availability

The original contributions presented in this study are included in the article. Further inquiries can be directed to the corresponding author.
